# A broadband terahertz ultrathin multi-focus lens

**DOI:** 10.1038/srep28800

**Published:** 2016-06-27

**Authors:** Jingwen He, Jiasheng Ye, Xinke Wang, Qiang Kan, Yan Zhang

**Affiliations:** 1 Department of Physics, Harbin Institute of Technology, Harbin, 150001, P.R. China; 2 Department of Physics, Capital Normal University, Beijing Key Laboratory of Metamaterials and Devices, and Key Laboratory of Terahertz Optoelectronics, Ministry of Education, Beijing, 100048, P.R. China; 3 State Key Laboratory for Integrated Optoelectronics Institute of Semiconductors Chinese Academy of Sciences, Beijing, 100083, P.R. China

## Abstract

Ultrathin transmission metasurface devices are designed on the basis of the Yang-Gu amplitude-phase retrieval algorithm for focusing the terahertz (THz) radiation into four or nine spots with focal spacing of 2 or 3 mm at a frequency of 0.8 THz. The focal properties are experimentally investigated in detail, and the results agree well with the theoretical expectations. The designed THz multi-focus lens (TMFL) demonstrates a good focusing function over a broad frequency range from 0.3 to 1.1 THz. As a transmission-type device based on metasurface, the diffraction efficiency of the TMFL can be as high as 33.92% at the designed frequency. The imaging function of the TMFL is also demonstrated experimentally and clear images are obtained. The proposed method produces an ultrathin, low-cost, and broadband multi-focus lens for THz-band application

Multiple focusing and imaging techniques are widely used in the fields of optical communication^1^, optical manufacture, photoelectric detection, optical computing and optical information processing^2^. For example, in the photoelectric detection, the microlens array integrated with the charge coupled device (CCD) cameras is an effective method to improve the photosensitivity of the CCD sensor^3^,^4^. In optical computing and optical information process, many types of parallel processing have been proposed to improve the processing. In parallel processing, there are many occasions where the performance of many miniature duplicate images of the input pattern is required^5^,^6^. Many methods are available to perform multiple focusing and imaging in the visible light, including spatial filters^7^, planar computer-generated hologram arrays, planar microlens arrays^6^, and Dammann gratings^8^,^9^. However, compared with the working wavelength, the conventional optical elements are very bulky. The emergence of the metasurface has greatly reduced the volume of optical devices and has drawn widespread research attention. The metasurface can realize arbitrary values of effective permittivity and permeability that do not exist in the natural world^10^,^11^,^12^,^13^. Since Yu *et al*.^14^ modulated the phase and amplitude of a cross-polarized scattered field using a specially designed metasurface, optical elements based on the metasurface have experienced rapid growth in recent years^15^,^16^,^17^. In 2014, Chen *et al*. designed an ultrathin broadband beam shaper based on a metasurface to obtain four foci in visible and near-infrared light range^18^. However, the proposed beam shaper requires that the incident light is circularly polarized and its diffraction efficiency is only 2%.

In the terahertz (THz) region, the corresponding wavelengths are located in the range of 30–3000 μm, which is much larger than the wavelength of visible light. Due to the lack of natural materials with high transmittance in the THz region, the THz devices are developed relatively slowly. The traditional THz devices are mainly made of high resistance silicon and polytetrafluoroethylene, and the volume of these devices is relatively large which limits the ongoing development of THz technology. Increasing numbers of THz devices based on the metasurface are being designed and applied, e.g., the THz waveplate^19^,^20^,^21^,^22^, the THz ultrathin lens^23^, the THz hologram plate^23^,^24^, and the THz vortex plate^25^. In order to promote the development of the THz cameras, THz communication, THz interconnection and storage systems, multiple focusing and imaging devices will be need. Several works on multiple focusing in the THz region have also been reported in recent years. In 2015, a holographic reflection metasurface device has been proposed for focusing the THz radiation into four spots^26^, and its diffraction efficiency can reach to 80%. Although a high efficiency can be easily obtained by a reflective-type metasurface, the transmission-type devices are essential in practice. Unfortunately, the transmission-type metasurface devices still suffer from low efficiency in the visible light range due to the ohmic losses, and efficiencies of these ultrathin metasurface devices are not more than 3%^18^,^23^,^27^,^28^. Wang *et al*. proposed a transmission-type metasurface device (a broadband flat-lens array) for multiple focusing in the THz region^29^. However, the designed flat-lens array area is large, and its size depends on the number of flat lenses. Meanwhile, the intensities of the foci are not uniform and most of the energy is concentrated at the center focus. To date, the transmission-type devices for multiple focusing applications with uniform intensity, multiple imaging capabilities and high efficiency have not been realized in the THz region.

In this paper, ultrathin multi-focus lenses based on the metasurface that provides multiple focusing and imaging capabilities with uniform intensity in the THz region are designed and fabricated. The Yang-Gu amplitude-phase retrieval algorithm is used to design the phase distribution of the THz multi-focus lens (TMFL). The feasibility of the proposed TMFL is also investigated using a THz holographic imaging system^30^,^31^. The focal properties of the TMFL, including the focal length, the focal depth, the focal spot size, and the diffraction efficiency, are investigated in detail. This designed TMFL performs over a broad frequency band of 0.3–1.1 THz, and its diffraction efficiency can reach to 33.92% at the designed frequency. This type of transmission metasurface serves as an attractive alternative to conventional diffractive optical elements (DOEs) based on its special performances of broadband operation, small area, ease of fabrication, and low cost.

## Theoretical Design

**Figure 1:**
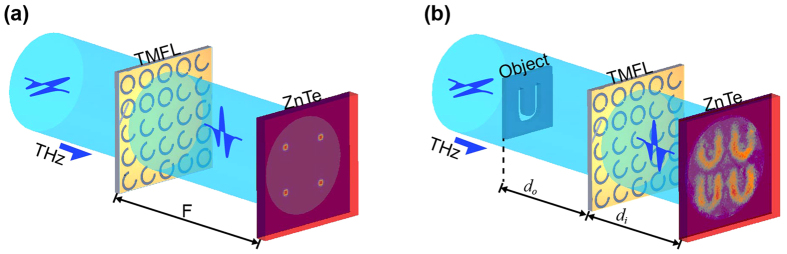
Schematic setup for focusing and imaging by using the designed TMFL. (**a**) Schematic view of multiple focusing. (**b**) Schematic view of multiple imaging. The parameter *F* represents the focal length of the designed TMFL; 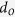 and 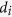 are the object and image distances in the imaging system, respectively.

**Figure 2:**
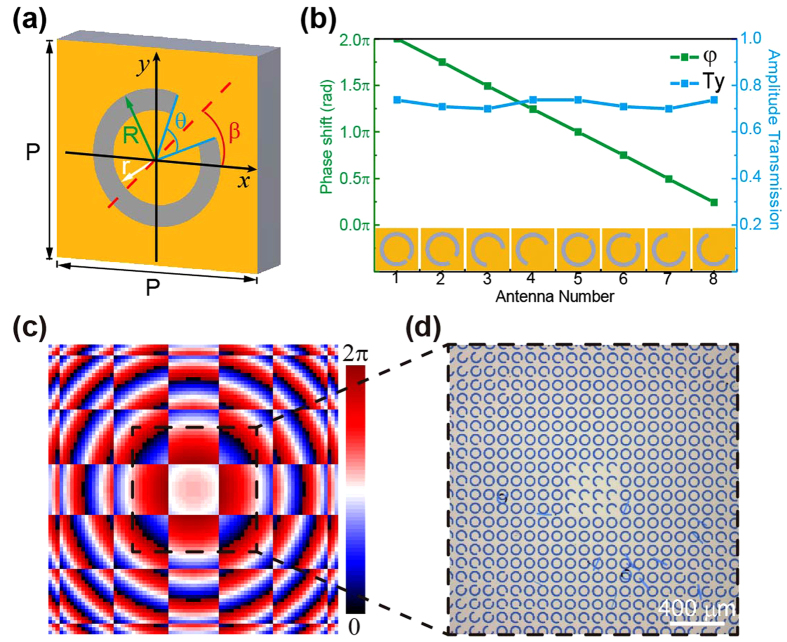
TMFL design. (**a**) Schematic view of C-shaped slot antenna. (**b**) Simulated amplitudes and phases of the cross-polarized scattered wave emitted by the eight selected antennas. (**c**) Quantized phase distribution of the TMFL for four focal spots with focal spacing of 2 mm and a focal length of 8 mm at 0.8 THz, which is calculated using the Yang-Gu algorithm. (**d**) Photograph of the central part of the designed TMFL sample.

The designed TMFL can provide multiple focusing (as shown in Fig. 1a) and multiple imaging (as shown in Fig. 1b) capabilities. It consists of C-shaped slot antenna units with various opening angles, and these antenna units are used to modulate the phase of the cross-polarized wave. When a linearly-polarized electromagnetic wave impinges on an antenna unit, both “symmetric” and “antisymmetric” modes can be supported. According to the theory proposed by Cappasso, when a cross-polarized field is scattered by these two modes, its amplitude and phase can be determined using the geometry parameters of the antennas^14^,^32^,^33^. Based on this principle, a series of C-shaped slot antennas are designed as the phase modulation units. Slot antennas can be used to block direct transmission and thus avoid any disturbances being caused by the incident radiation. Each C-shaped slot antenna consists of a gold slot and a substrate, as shown in Fig. 2a. The slot is fabricated in a 100-nm-thick gold film that was deposited on a double-side polished high-resistivity silicon substrate (which is 500 μm thick). The parameters of the antenna unit are marked out in Fig. 2a. The azimuth angle (*β*) of the C-shaped slot is defined as the angle between the axis of symmetry and the x-axis. *R* and *r* are the outer and inner radius of the C-shaped slot, respectively; and *θ* is the opening angle of the split. *P* is the period of the antenna unit. To realize the phase change of the cross-polarized wave from 0 to 2π with identical amplitudes at the operating frequency of 0.8 THz, eight antenna units are selected, as shown in Fig. 2b. The phase of the cross-polarized scattered wave that is emitted by antenna No. 1 is defined as the reference. It can be clearly seen that the cross-polarized scattered fields have almost identical amplitudes and a constant phase difference of π/4 among the eight antennas. The antenna unit parameters are chosen as follows: *P* = 100 μm, *R* = 40 μm, and *r *= 30 μm. For the antenna units numbered from 1 to 4, the azimuth angle is *β* = −45°, and the split opening angles are *θ* = 10°, 35°,100°, 132°, respectively. For the antenna units numbered from 5 to 8, the azimuth angle is *β* = 45°, and the split opening angles are *θ* = 10°, 35°,100°, 132°, respectively. The simulated results in Fig. 2b are calculated using a commercial simulation software (FDTD Solutions) based on the finite-difference time-domain (FDTD) method. In the simulation, the antenna unit shown in Fig. 2a is used as the model. An x-polarized plane wave with an operating frequency range from 0.2 to 1.5 THz is used to impinge on the bottom of the substrate. Periodic boundary conditions are applied in both x and y directions, while perfectly matched layers are applied in the propagation direction. The refractive index of the high-resistivity silicon substrate is 3.4 in this waveband.

To obtain the expected phase profile of the TMFL, the Yang-Gu amplitude-phase retrieval algorithm^34^, which has been widely used in the design of diffractive optical elements, is employed. It is based on a feedback iterative process. In each iteration, the calculated field distribution on the focal plane is compared with the target field until the calculated field is very close to the target value. At this time, the phase distribution on the input plane is just the required phase 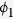 (see the first subsection in Methods). The obtained phase values are then quantized into eight values, ranging from 0 to 2π, and the eight antennas mentioned earlier are used to fill the corresponding positions. The quantized phase distribution (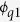) of the designed TMFL for four focal spots with focal spacing of 2 mm and a focal length of 8 mm at 0.8 THz is shown in Fig. 2c and a photograph of the central part is shown in Fig. 2d. The entire TMFL is composed of 100 × 100 antenna units in a 10 × 10 mm^2^ area.

## Results and Discussions

### Focusing of the TMFL

**Figure 3:**
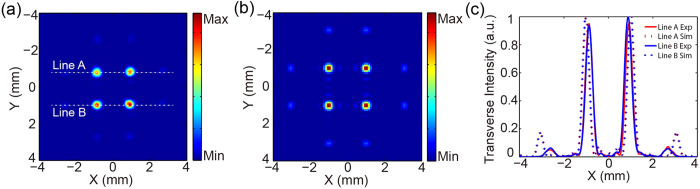
Focusing results of the designed TMFL. Experimental (**a**) and theoretical (**b**) intensity distributions on the focal plane at 0.8 THz, respectively. (**c**) Transverse intensity profiles along the dashed lines in (**a**) on the focal plane. The red and blue curves represent the experimental transverse intensities of Line A and Line B, respectively. The dashed lines indicate the theoretical transverse intensities.

Figure 3a displays the intensity distribution measured on the focal plane. Four focal spots can be observed and the distance between pairs of neighboring spots along the x and y directions is 2 mm. For comparing with the experimental result, the propagation of the initial field (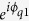) is calculated using the Fresnel diffraction integral with a propagation distance of 8 mm. The calculated intensity distribution on the focal plane at 0.8 THz is shown in Fig. 3b. To demonstrate the properties of the TMFL more clearly, the theoretical and experimental transverse intensity distributions along the dashed lines marked in Fig. 3a are illustrated in Fig. 3c using solid and dashed lines, respectively. Both theoretical and experimental transverse intensity distributions are normalized by their respective maximum values. The blue and red curves represent the intensity distributions that are extracted along Line A and Line B, respectively. Each focal spot shows a Gaussian shape with a full-width-at-half-maximum of 352 μm, and the intensities of the four focal spots are almost equal although the intensity of the spot along Line B is little higher than the intensities of the others. This difference is mainly caused by fabrication errors. The experimental result shows a basic agreement with the theoretical expectation.

### Broadband focusing properties of the TMFL

**Figure 4:**
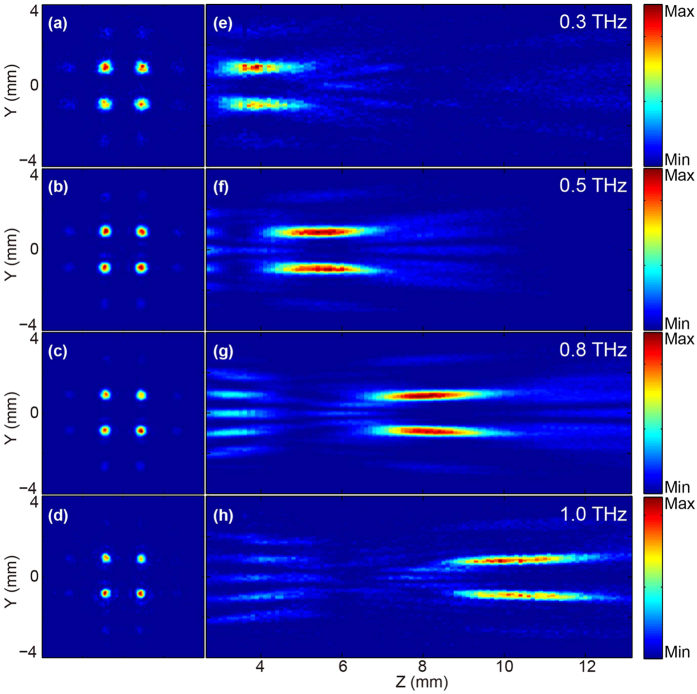
Experimental intensity distributions at different frequencies. (**a**–**d**) Intensity distributions of *Ey* in focal planes at 0.3, 0.5, 0.8, and 1.0 THz, respectively. (**e**–**h**) Longitudinal intensity distributions of the cross-polarized field *Ey* on the yz plane at 0.3, 0.5, 0.8, and 1.0 THz, respectively.

To investigate the focusing properties of the TMFL during the propagation, a Z-scan measurement is performed by simply moving the TMFL along the Z-axis. The distance between the TMFL and the probe crystal (ZnTe in Fig. 1) is varied from 3 mm to 13 mm with a scan resolution of 0.1 mm. The focal intensity distributions on each focal plane (the xy plane) at the frequencies of 0.3, 0.5, 0.8, 1.0 THz are shown in Fig. 4a–d, respectively. Figure 4e–h illustrate the corresponding longitudinal intensity distributions on the yz plane during the propagation; these distributions are extracted along the line with two left foci (x = −1 mm). The TMFL demonstrates a good focusing property over a broad frequency band from 0.3 to 1.1 THz. Clearly, the actual focal length and the focal depth increases with increasing frequency, while the focal spot size decreases. The values of the actual focal length, the focal depth, and the focal spot size are shown in Table 1 in detail. With the frequency increases from 0.3 to 1.1 THz, the focal length increases from 3.5 to 11.3 mm, and the focal depth increases from 1.2 mm to 2.8 mm, while the focal spot size decreases from 448 μm to 256 μm.

**Figure 5:**
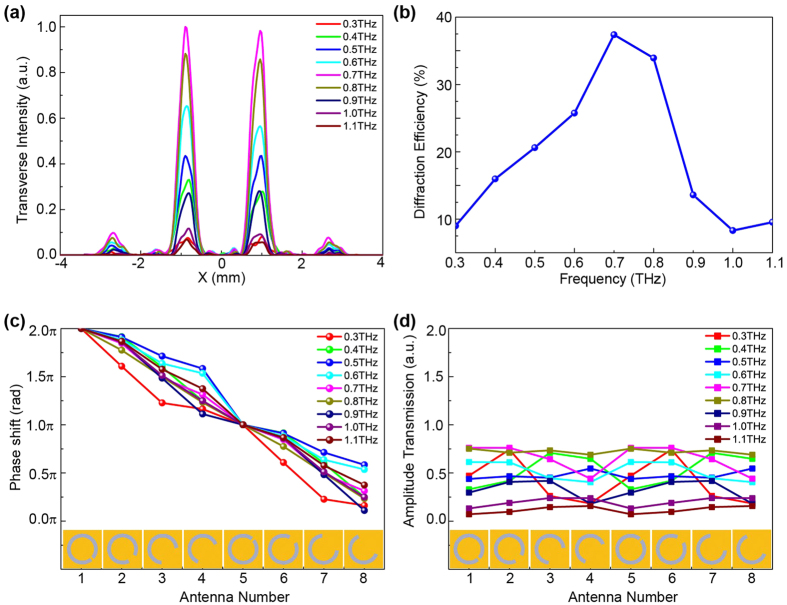
Focusing performances of the designed TMFL at different frequencies. (**a**) Experimental transverse intensity profiles along the two upper foci of each focal plane over the frequency range from 0.3 to 1.1 THz. (**b**) Measured diffraction efficiency with respect to frequency in the range from 0.3 to 1.1 THz. (**c**,**d**) Phase shift and amplitude transmission of the cross-polarized scattered wave, respectively, with respect to the eight antennas operating at different frequencies.

Another important property for a diffractive optical element is its diffraction efficiency, which is defined as the ratio of the intensity on the focal plane to the intensity of the incident wave at the same frequency. The experimental transverse intensity distributions along the two upper foci on each focal plane with frequencies ranging from 0.3 to 1.1 THz are shown in Fig. 5a. For better comparison, the focal intensities are normalized by the maximum value. The focal spacing is maintained at a constant 2 mm for all frequencies. The peak intensities of the foci for 0.7 THz are higher than those for other frequencies. When the frequency deviates from 0.7 THz, the focal intensity weakens. The focal intensity is mainly determined by the energy of the incident light and the diffraction efficiency of the TMFL. For our THz source, the energy at 0.7 THz is the largest, and the energy gradually decreases as the frequency deviates from the 0.7 THz. The diffraction efficiencies with respect to the frequencies are shown in Fig. 5b, and the measured diffraction efficiencies are also listed in Table 1. The diffraction efficiency of the TMFL is 33.92% for the designed frequency of 0.8 THz. For a transmission-type metasurface device, this diffraction efficiency is higher than that in the previously reported work^18^, in which the efficiency value is only 2%. The maximum diffraction efficiency is 37.39% for the frequency of 0.7 THz. For other frequencies, the diffraction efficiencies are lower than that for 0.7 THz. These TMFL properties are attributed to the selected antennas. In the THz region, gold can be treated as perfect electric conductor, thus the ohmic loss is negligible^35^. Therefore, a high diffraction efficiency can be reached. To illustrate this point, the phase shift and amplitude transmission of the cross-polarized wave *Ey* of these eight antennas for various frequencies are also simulated, the results are shown in Fig. 5c,d. For the optimal frequency of 0.8 THz, the phase of the cross-polarized wave *Ey* can be modulated from 0 to 2π at an almost constant gradient of π/4, and the amplitude transmission is quite uniform with an amplitude transmittance of 0.75. However, when operating away from the optimal frequency, the phase responses do not follow a perfect linear profile and the amplitude transmissions are not uniform for all eight antennas. Although the amplitude transmission for each antenna are different, the phase can be modulated almost over the entire range from 0 to 2π by the eight antennas over a broad frequency range of 0.3–1.1 THz. Therefore, the designed TMFL demonstrates suitable broadband performance. The amplitude transmission of the cross-polarized wave *Ey* is almost at a maximum at 0.7 and 0.8 THz, as shown in Fig. 5d. The amplitude transmission decreases with increasing deviation from these two frequencies. This may lead to the reduction of the diffraction efficiency for the TMFL.

### Imaging of the TMFL

**Figure 6:**
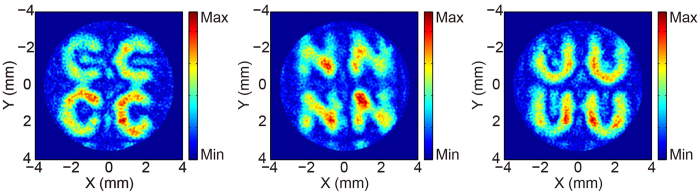
Images of three letters at 0.8 THz: “C”, “N”, and “U”.

The imaging function of the lens is also quite important in addition to its focusing function. To demonstrate the imaging performance of the designed TMFL, an imaging experiment is performed. The objects are three letter patterns (C, N, and U) which have been milled on a stainless steel sheet. The size of each letter is approximately 4 × 4 mm^2^ and the slot width is about 1 mm. The object and image distances 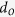 and 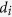 can be determined using the object-image formula 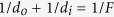, where 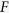 is the focal length of the lens as shown in Fig. 1. In the experiment, the focal length of the TMFL is designed to be *F* = 8 mm, and the object distance 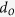 is set as 24 mm. Therefore, the image distance 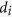 is 12 mm. The magnification *m* of the image also can be determined by using the relation 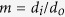, which gives a result of 0.5. The intensity distributions on the image plane for 0.8 THz are shown in Fig. 6. Because the TMFL is designed to have four focal points, four images are obtained with sizes of 2 × 2 mm^2^. All three letter pattern images are clear, which demonstrates the quality of the imaging performance of the designed TMFL.

### Designed TMFL with different focal spacing and focal numbers

**Figure 7:**
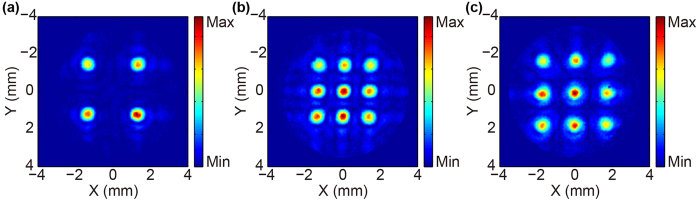
Experimental focal intensity distributions for TMFLs with different parameters operating at 0.8 THz. (**a**) Focal intensity distribution for four foci with focal spacing of 3 mm. (**b**,**c**) Focal intensity distributions for nine foci with focal spacing of 1.5 mm and 2 mm, respectively.

To further demonstrate the validity of the proposed TMFL design method, three other TMFLs with different focal spacing and focal numbers are designed and fabricated. The designed focal lengths of these TMFLs are all also set at 8 mm. The experimental results for the intensity distributions in the focal planes of these TMFLs are shown in Fig. 7. The focal intensity distribution for four foci with focal spacing of 3 mm is displayed in Fig. 7a. The focal intensity distributions for nine foci with focal spacing of 1.5 mm and 2 mm are shown in Fig. 7b,c, respectively. These results indicate that the TMFLs with other focal numbers and focal spacing can also be realized using this method.

## Conclusions

In conclusion, we designed several ultrathin TMFLs which contain a series of C-shaped slot antenna units based on the Yang-Gu algorithm. The experimentally measured intensity distributions on the focal plane show good agreement with the designed objective. The device exhibits broadband properties with a bandwidth of 800 GHz and the diffraction efficiency can reach to 33.92% at the designed frequency. The designed TMFL also demonstrates good imaging performances. In addition, we also designed and verified a TMFL with four foci with focal spacing of 3 mm and TMFLs with nine foci with focal spacing of 1.5 mm and 2 mm. We believe that this work will be valuable for the application of these devices to THz imaging and communications.

## Methods

### Yang-Gu amplitude-phase retrieval algorithm

The phase distribution of the TMFL is designed using the Yang-Gu amplitude-phase retrieval algorithm. The TMFL is fixed in the input plane P1. The output plane P2 is located at 8 mm away from the TMFL. A plane wave is transmitted through the TMFL and assumed to be focused in the plane P2 with four focal spots. Based on the Yang-Gu algorithm, the complex field distribution on the input plane can be written as:


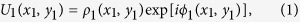


and the target field distribution on the output plane is given by


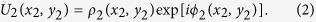


where 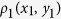 and 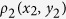 are the amplitude distributions on the input and output planes, respectively; 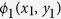 and 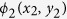 are the phase distributions.

The relationship between 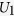 and 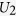 can be described as follows:


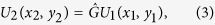



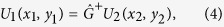


where 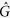 denotes the Fresnel diffraction transform function and 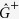 denotes the inverse transformation. The phase distributions on planes P2 and P1 can be obtained as follows:


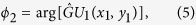



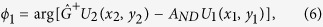


with


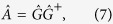



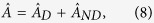


where 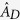
(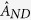) is the diagonal (off-diagonal) element of 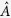


The optimal TMFL design based on the Yang-Gu algorithm is realized by using an iterative process. First, the initial amplitude and phase of 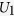 are given by 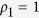, and 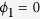; second, the phase distribution of 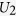 is calculated using Eq. (5); third, the calculated 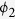 is substituted into Eq. (2) to obtain a new 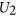, where the amplitude distribution of the target 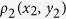 is known; fourth, a new 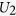 is then plugged into Eq. (6) to get a new 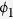; fifth, steps 2, 3, and 4 are repeated until the calculated 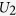 value is close to the target value, and the difference accuracy is given by


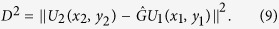


Finally, the phase distribution of the TMFL 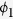 can be obtained.

### Measurement method

**Figure 8:**
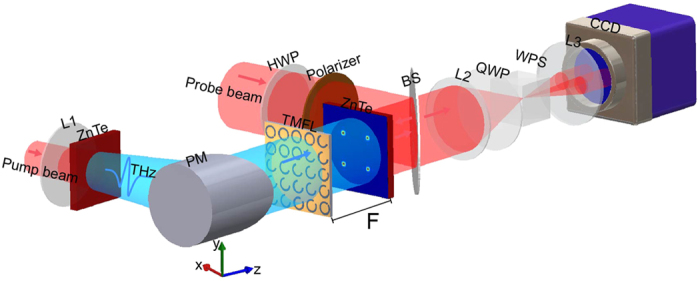
Experimental setup for characterization of the performance of the designed TMFL. (HWP: half-wave plate; PM: parabolic mirror; BS: beam spliter; QWP: quarter-wave plate; WP: Wollaston prism).

In the experiment, a THz holographic imaging system^30^,^31^ is used to characterize the focusing performance of the TMFL, and part of the experimental setup is shown in Fig. 8. The light source is a 100 fs ultrashort laser pulse with an 8-mm spot diameter, an 800-nm central wavelength and a 1-kHz repetition rate provided by a Ti: sapphire regenerative amplifier. The laser beam, which has an average power of 900 mW, is divided into two parts, which are the pump (880 mW) and probe (20 mW) beams used for generation and detection of the THz waves, respectively. The pump beam is diverged using a concave lens (L1) with a focal length of 50 mm. A <110> ZnTe crystal is placed at the rear of the concave lens to generate a terahertz beam based on optical rectification. A parabolic mirror with a focal length of 150 mm is then used to collimate the THz wave. Then, a horizontal THz beam (*x*-polarized: *Ex*) with a diameter of 24 mm impinges on the TMFL, and the scattered vertical THz beam (*y*-polarized: *Ey*) is detected by another <110> ZnTe crystal, which is located 8 mm away from the TMFL. In the probe optical path, a half-wave plate (HWP) and a polarizer are used to modulate the probe beam polarization. Then, the probe beam with the vertical polarization is reflected by a non-polarization beam splitter (BS) (T:R = 5:5) towards the sensor crystal. In our experiment, the <001> axis of the sensor crystal is set perpendicular to the vertical direction to enable it to measure the THz vertical polarization component (*Ey*)^30^. In the sensor crystal, the probe beam polarization is modulated by the THz field based on the Pockels effect, and the reflected probe beam is then captured by the imaging unit. The imaging unit consists of a 4f system (two convex lenses, L2 and L3), a quarter-wave plate (QWP), a Wollaston prism (WP), and a charge-coupled device (CCD) camera. The WP is used to split the probe beam into two beams with orthogonal polarizations, and the two images of the sensor crystal are then projected onto the CCD camera by the 4f system. The THz complex field can be extracted using the balanced electro-optic detection technique^30^,^31^. By varying the optical path difference between the THz beam and the probe beam, 100 temporal images are captured at each time delay, and the time window is 17 ps. The intensity and phase information at the different frequencies can be extracted by performing Fourier transformations on the temporal signals at each pixel.

## Additional Information

**How to cite this article**: He, J. *et al*. A broadband terahertz ultrathin multi-focus lens. *Sci. Rep.* **6**, 28800; doi: 10.1038/srep28800 (2016).

## Figures and Tables

**Table 1:** Focusing properties of the TMFL at different frequencies.

Frequency (THz)	Focal length (mm)	Focal depth (mm)	Focal spot size (μm)	Diffraction efficiency (%)
0.3	3.5	1.2	448	8.99
0.4	4.6	1.8	416	15.97
0.5	5.5	2.1	384	20.61
0.6	6.4	2.0	352	25.75
0.7	7.3	2.4	352	37.39
0.8	8.0	2.4	352	33.92
0.9	8.9	2.3	352	13.60
1.0	10.0	2.8	320	8.31
1.1	11.3	2.8	256	9.54
